# Bait uptake by wild badgers and its implications for oral vaccination against tuberculosis

**DOI:** 10.1371/journal.pone.0206136

**Published:** 2018-11-09

**Authors:** Stephen P. Carter, Andrew Robertson, Kate L. Palphramand, Mark A. Chambers, Robbie A. McDonald, Richard J. Delahay

**Affiliations:** 1 National Wildlife Management Centre, Animal and Plant Health Agency, Woodchester Park, Gloucestershire, United Kingdom; 2 Vincent Wildlife Trust, Eastnor, Ledbury, Herefordshire, United Kingdom; 3 Environment and Sustainability Institute, University of Exeter, Penryn, Cornwall, United Kingdom; 4 Department of Bacteriology, Animal and Plant Health Agency, Weybridge, Woodham Lane, New Haw, Surrey, United Kingdom; CEFE, FRANCE

## Abstract

The deployment of baits containing vaccines or toxins has been used successfully in the management of wildlife populations, including for disease control. Optimisation of deployment strategies seeks to maximise uptake by the targeted population whilst ensuring cost-effectiveness. Tuberculosis (TB) caused by infection with *Mycobacterium bovis* affects a broad range of mammalian hosts across the globe, including cattle, wildlife and humans. The control of TB in cattle in the UK and Republic of Ireland is hampered by persistent infection in European badgers (*Meles meles*). The present study aimed to determine the best strategy for maximising uptake of an oral vaccine by wild badgers, using a surrogate novel bait deployed at 40 badger social groups. Baits contained a blood-borne biomarker (Iophenoxic Acid, IPA) in order to measure consumption in badgers subsequently cage trapped at targeted setts. Evidence for the consumption of bait was found in 83% (199/240) of captured badgers. The probability that badgers had consumed at least one bait (IPA >10 μg ml^-1^) was significantly higher following deployment in spring than in summer. Lower uptake amongst social groups where more badgers were captured, suggested competition for baits. The probability of bait consumption was significantly higher at groups where main and outlier setts were provided with baits than at those where outliers were present but not baited. Badgers captured 10–14 days post bait feeding had significantly higher levels of bait uptake compared to those caught 24–28 days later. Uptake rates did not vary significantly in relation to badger age and whether bait was placed above ground or down setts. This study suggests that high levels of bait uptake can be achieved in wild badger populations and identifies factors influencing the potential success of different deployment strategies. The implications for the development of an oral badger vaccine are discussed.

## Introduction

The deployment of baits is carried out routinely in many countries to deliver vaccines, toxicants, fertility control agents or medication to wildlife populations [1, 2]. Examples of the successful use of bait deployment for wildlife management include the widespread aerial delivery of toxic baits for reducing densities of introduced brushtail possums (*Trichosurus vulpecula*) in New Zealand [3] and of oral vaccine baits to control sylvatic rabies in the USA and Europe [4]. However, targeted bait deployment may be required to reflect the distribution of the target species, geographic constraints and concerns over uptake by non-target species.

Tuberculosis (TB; *Mycobacterium bovis* infection), is a globally important animal disease and the most serious health threat to the cattle industry in the UK and the Republic of Ireland. The associated annual costs (including testing, research and compensation) to the UK government are approx. £100 million [5]. European badgers (*Meles meles*) are involved in the maintenance and transmission of *M*. *bovis* to cattle in both countries [6–9]. Hence there has been considerable research interest in the development of TB vaccines for both badgers and cattle. Parenteral and oral delivery of BCG (Bacillus Calmette-Guérin) to captive badgers has been shown to significantly reduce the severity of disease associated with experimental *M*. *bovis* challenge [10–13]. Furthermore, field trials of both parenteral [14] and oral [15] delivery have indicated a reduced likelihood of TB infection in both vaccinated and unvaccinated animals; the latter by means of a herd immunity effect. Since licensing of the vaccine in 2010 several thousand doses of BadgerBCG have been delivered (through live-trapping and injection) in England and Wales by government agencies, and by voluntary and community sector organisations (APHA unpublished data). However, as the delivery of BadgerBCG by cage-trapping and injection is labour intensive, there has been considerable investment in the development of a candidate oral bait for BCG delivery [16–18] which may offer a more cost-effective means of vaccinating badgers at a wider scale. The potential contribution of an oral vaccine to TB control was highlighted by a recent study which provided field evidence for a significant protective effect of oral BCG administration in a wild badger population [15].

The cost-effective delivery of a vaccine bait to wild badger populations will require development of a deployment strategy that reflects their spatial organisation and behaviour. Across much of the TB affected areas of the UK, badgers are found at moderate to high densities, typically comprising social groups of between six and eight individuals occupying a group territory [19]. Most territories in high-density populations contain between three and six underground dens (setts) usually comprising one main sett which serves as the primary year-round residence and other smaller (outlier) setts that tend to be occupied less frequently [19, 20]. Badger setts therefore serve as a useful focus for vaccine delivery, although the added benefits (or otherwise) of deploying baits at outlier setts in addition to main setts are not yet known. It is also not clear whether greater benefits would be achieved through the dispersal of baits above ground (to avoid monopolisation by one or two dominant animals) versus simply deploying them down active badger sett entrances. The seasonal effects of bait uptake are also poorly understood, with the limited data available suggesting that relatively low rates of uptake in winter are followed by substantially higher rates in early spring (February) and early summer (June) [21]. The need to target each new cohort of susceptible badger cubs in any vaccination programme makes it logical to deploy bait soon after cubs have been weaned, which is typically from May to June onwards in southern England [19]. In the present study we investigated uptake of baits designed for oral vaccine deployment at 40 badger social groups. Baits incorporated a blood borne biomarker (iophenoxic acid) so that their consumption could be detected in subsequently captured animals. By varying the deployment protocol we aimed to determine whether bait uptake in badgers varied in relation to season (by comparing uptake in late spring (May/June) to mid-summer (July/August)), bait placement (by deploying baits either into burrow entrances or above ground under tiles) and deployment strategy (by placing baits at main setts only or at all setts associated with a social group).

## Methods

### Ethics statement

All animal trapping and associated procedures were covered by licences issued by Natural England and the Home Office, following approval by an internal ethical review process at The Food and Environment Research Agency and Animal Health and Veterinary Laboratories Agency (now the Animal and Plant Health Agency).

### Study areas and populations

The study was carried out in three geographically distinct regions (Cirencester, Bath and Langford) in two counties in southwest England (Gloucestershire and Somerset) between May and August 2010. Study areas were in landscapes of typically moderate to high badger density (approx. 3 to 8 badgers per km^2^) [22] within a TB-endemic part of England, and were therefore deemed representative of where oral vaccination of badgers could potentially take place. The selected areas contained badger populations that had not previously been studied to avoid any bias associated with prior experience of baits. The study areas were typically comprised of mixed woodland and agricultural land, and covered a total of 862 km^2^.

We had no prior knowledge of the territorial configuration of badger groups in our study populations and could not determine this using the standard approach as this involves bait deployment [23]. Surveys for badger field signs were conducted by experienced field staff who identified and differentiated badger main setts from outlier setts. We assigned all outlier setts showing signs of badger activity to a social group if they fell within a 300 m radius of the main sett. Using unpublished data from a comparable badger population in Gloucestershire [14], we considered 300 m to be an appropriate distance from the main sett within which we should capture the majority of associated outlying setts whilst excluding outlying setts from neighbouring social groups. To minimise the risks of non-independence of selected badger social groups, targeted main setts were at least 2 km apart. In total, 40 social groups (each containing one main sett) were identified for inclusion in the study.

### Baits and biomarkers

At the time of the present study a defined candidate vaccine bait had not yet been identified and so we used a bait comprised of peanuts and syrup that is known to be highly attractive to badgers [23]. Each bait portion consisted of 100 g of peanuts and syrup containing 80 mg of Propyl-Iophenoxic Acid (P-IPA, hereafter referred to as IPA).

### Bait deployment

To investigate seasonal variation in uptake, baits were deployed at badger setts in either spring (n = 20, bait deployed 17^th^ May– 7^th^ June), or summer (n = 20, bait deployed 12^th^ July to 2^nd^ August Table 1). Autumn and winter were not considered in this study as it will be preferable to target cubs with a vaccine earlier in the year prior to them potentially being exposed to TB. Natural food availability is also lowest in spring and summer, so badgers may be more likely to consume baits. For logistical reasons it was not possible to deploy baits at all setts simultaneously in each season; instead the 20 setts were randomly split into three roughly equal groups at which feeding commenced one week apart. Baits were deployed at setts in the late afternoon (to minimise exposure to rainfall and non-target wildlife most likely to be active during the day) according to the particular treatment combination assigned to each social group. Main setts received five baits (day 1), ten baits (day 2), 15 baits (day 3 onwards) for 12 consecutive days (total of 165 bait portions or 16.5 kg of bait per main sett). The rationale for incrementally increasing the number of baits initially was to acclimatise the badgers to the novel food source, as described previously [23]. Outlying setts being typically smaller than main setts and with fewer badgers likely to be in residence, were fed fewer baits and the number fed remained constant from day 1. Outliers (in groups assigned the “all setts” treatment) were fed five baits daily for 12 consecutive days. Baits were either placed above ground (n = 20, 9 in spring, 11 in summer) in a shallow depression beneath a 20 x 20 cm (~2.5 kg) floor tile (providing protection from weather and non-target species) located near sett entrances or on runs (paths) within 20 m of sett entrances, or below ground (n = 20, 11 in spring, 9 in summer) by simply rolling into active sett entrances, distributed as evenly as possible amongst them. Hence, where there were two active entrance holes at a sett, from day 3 onwards eight baits would be deployed down one and seven down the other. The appropriate number of new baits (dependent on day of feeding and type of sett) were deployed below ground each day, whilst bait under tiles was only replaced if ≥ 50% had been taken. For the groups receiving bait above ground, bait disappearance was recorded for eleven consecutive days and again at day 14 when all uneaten bait was removed. We also investigated whether uptake would depend on where baits were deployed (i.e. at which setts/burrows), by deploying baits either at main setts only (i.e. the main burrow) or at main setts and outlier setts (within 300 m). Badger social groups were spread among the three areas (Table 1) and were randomly assigned to a season (spring/summer) or bait placement group (above ground/below ground), such that the combinations of categories were approximately evenly split (Table A in S1 Appendix). Social groups were also randomly assigned a bait deployment treatment (main setts only/ main setts and outlier). However, the number of outlier setts was not considered (many groups had no outlier setts), therefore social groups had to be split into one of three categories (main setts only, outliers present and fed, outliers present but not fed), with most groups falling into the first two categories (Table 1).

**Table 1:** Details of variables and numbers of social groups (main setts) in each treatment category.

Variable	Treatment	Details	n
Bait placement	Below ground	Baits deployed down sett entrances	20
	Above ground	Baits placed under ceramic tiles	20
Deployment strategy	Main setts only	These groups had no outlier setts ≤300m from main sett, so bait only fed at main setts	24
	Main setts and outliers fed	These groups had active outlier setts ≤300m and all were fed bait in addition to main setts	12
	Main setts fed but outliers not	These groups had active outlier setts ≤300m but bait only fed at main setts	4
Season	Spring	Bait fed 17th May - 7th June	20
	Summer	Bait fed 12th July - 2nd August	20
Location	Bath	-	13
	Cirencester	-	11
	Langford	-	16
			

### Badger trapping and blood sampling

Badgers were trapped in two operations, 10–14 days and 24–28 days after bait deployment. In both instances steel mesh traps [24] were placed in the proximity of badger setts, pre-baited with peanuts (no syrup or biomarker) for 5–10 days and subsequently set to catch for two consecutive nights. Captured badgers were transported to a mobile sampling facility where they were anaesthetised by intra-muscular injection of a combination of ketamine hydrochloride (100 mg ml-1, VetalarTM V, Pharmacia & Upjohn, Crawley, UK), medetomidine hydrochloride (1 mg ml-1, Domitor, Pfizer, Sandwich, UK) and butorphanol tartrate (10 mg ml-1, Torbugesic, Fort Dodge Animal Health Ltd, Southampton, UK) at a ratio of 2:1:2 by volume respectively [25].All badgers were permanently marked by tattoo on the lower abdomen. The weight, sex, age (adult or cub) and body length of each animal was recorded and up to 17 ml of blood (dependent on body weight) was taken for biomarker detection. Blood samples were centrifuged to separate serum and stored immediately at -20° C. After sampling, captured badgers were allowed to recover before being returned to their point of capture and released.

### Detection of biomarker

Blood samples were analysed by LGC Ltd. (formerly the Laboratory of the Government Chemist: Teddington, UK) for the presence of IPA compounds by liquid chromatography-mass spectrometry (LC-MSMS). LC-MSMS has a limit of detection (LoD) for IPA in serum of 50 ng ml^-1^. Previous analyses of badger serum for IPA compounds classified individuals as having consumed at least some bait (which contained 10 mg per 100 g portion) if IPA levels were above 0.125 μg ml^-1^ [26]. In another study badgers consuming 10 mg of IPA had levels of ~2.5 μg ml^-1^ after 6 weeks, and detectable amounts up to 16 weeks [27]. In the current study each 100 g bait portion contained 80 mg of IPA and the potential time between bait consumption and capture in the current study was 11–40 days. Given the nature of the bait, it is possible that badgers consumed only part of a bait portion which would likely result in a positive IPA result (i.e. above zero). We chose a cut off of >10 μg ml^-1^ of IPA to confidently indicate consumption of bait. It is unclear whether this would relate to an entire single portion of bait, however, the primary aim of the study was to compare relative levels of uptake (percentage of individuals eating bait) in different treatment and demographic groups.

### Statistical analyses

In order to investigate which factors influenced the likelihood of bait uptake (i.e. an IPA result of ≥10 μg ml^-1^, binomial 0/1) by individual badgers we carried out a series of generalised linear mixed model analyses (logistic regression). Potential fixed effects in the models were age (adult or cub), sex, season (spring or summer), bait placement (under tiles or below ground), trapping session (1^st^ or 2^nd^), regional area (Bath, Cirencester, Langford) and putative group size (number of individuals trapped at a social group). Although trapping efficiency is unlikely to be 100%, the number of individuals caught acts as a proxy for group size and of competition for baits, assuming that greater numbers of badgers are caught at larger groups. Bait deployment strategy was included as a three-level factor (main setts only, outliers present but not fed and outliers present and fed) as some social groups had no active outlier setts, so it was necessary to differentiate between those that had no outliers and those that did (which were either fed bait or not). Two-way interactions between combinations of age, season, bait placement, group size and deployment strategy were also included. Social group (defined by the main sett identity) was included as a random effect. Although animals may have been captured and sampled more than once, only the IPA result from the first capture was included in the analyses.

For models of bait uptake, all potential combinations of explanatory variables were evaluated and ranked using Akaike’s information criterion (adjusted for small sample sizes; AICc). We then classified a top model set using a change in AICc (difference from the top model) cut off of ≤ 6, as this threshold provides 95% confidence that the most parsimonious model is included [28, 29]. Where multiple models were contained within this top set, average model coefficients were calculated and variables classed as having a consistent or significant effect if the 95% CI of the coefficients did not span zero [30]. All analyses were conducted in R (3.0.2, cran.org), mixed models were created using the package ‘lme4’ [31] while model comparison and averaging were conducted using the package ‘MuMIn’[32]. Paired t-tests and correlation analyses were used to investigate temporal differences in IPA values for recaptured badgers which were caught on the first and second trapping events.

## Results

### Bait uptake

**Fig 1:**
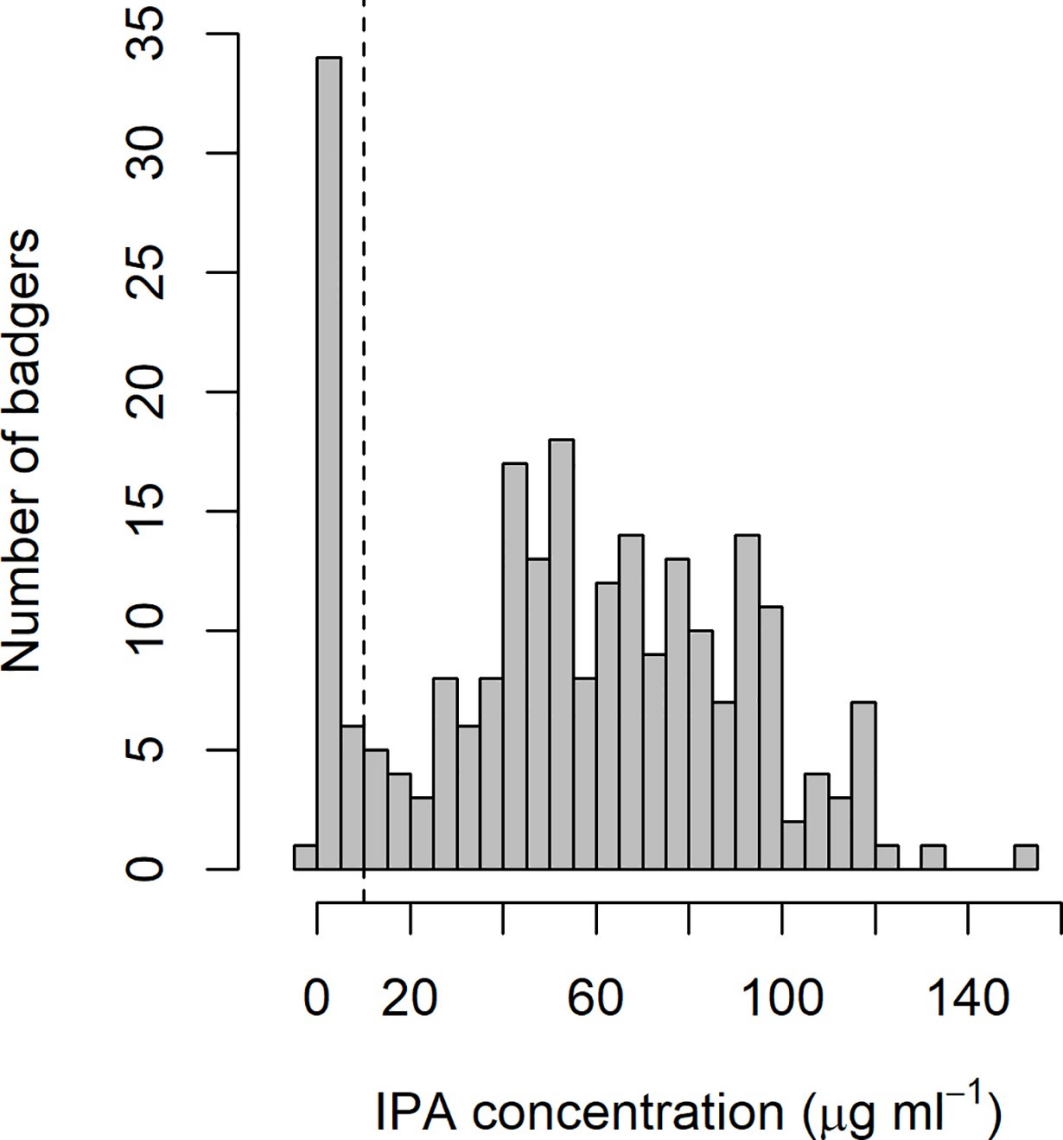
Distribution of IPA concentrations in the blood of 240 sampled badgers.).

**Fig 2:**
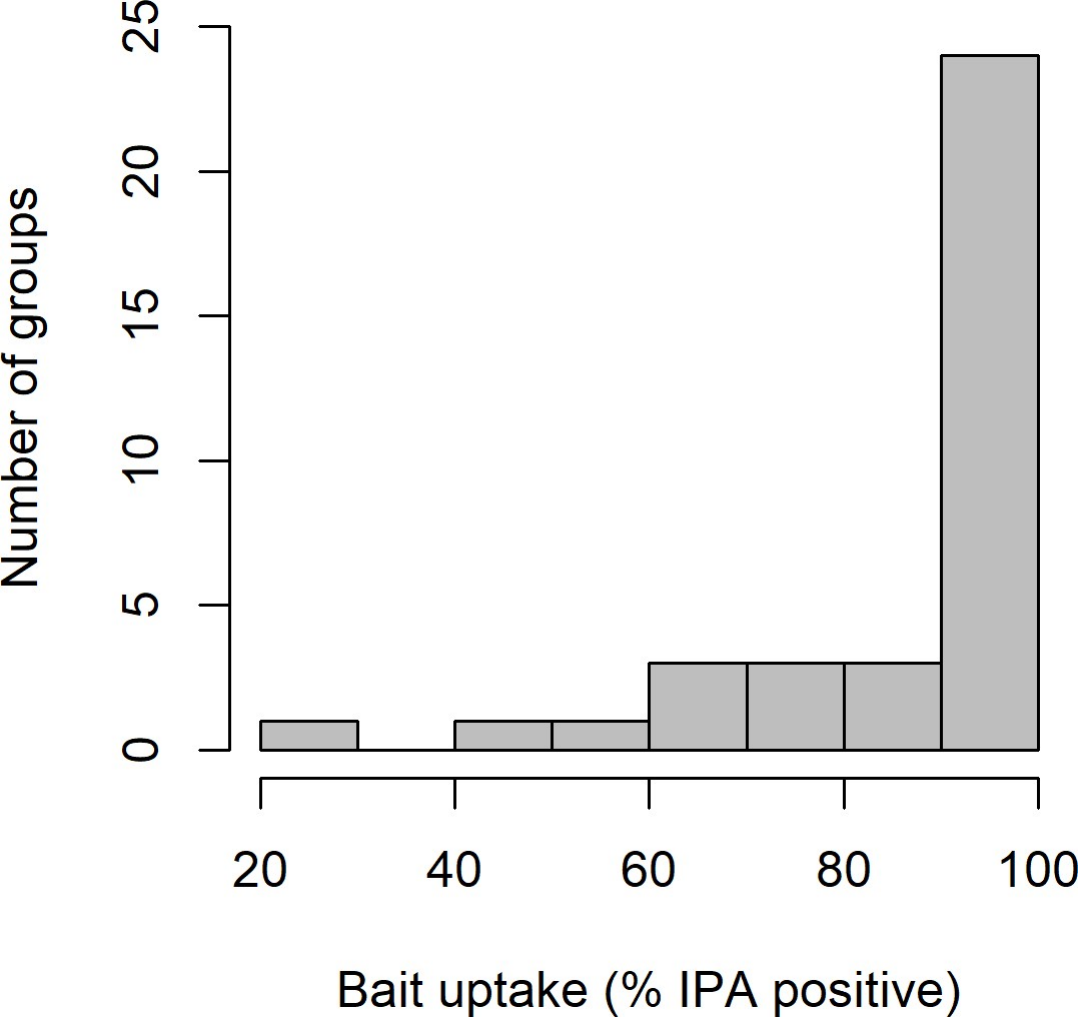
Variation in the percentage of badgers with>10 μg ml^-1^ IPA in blood amongst the 36 social groups where bait was fed and where animals were subsequently captured.

Across the two trapping sessions a total of 362 blood samples were obtained and successfully analysed from 240 individual badgers captured from 36 social groups. No badgers were trapped at the remaining four social groups. In total 186 adults were captured (97 in spring, 89 in summer) and 54 cubs (27 in spring and summer), which were spread relatively evenly among treatment categories (Figure A in S1 Appendix). The sex ratio of captured badgers was approximately equal (53% females, 47% males). Mean group size (number individuals trapped at each group) was 6.7 badgers (range = 1 to 29). This is slightly lower than the typical social group size in this habitat type (mean = 7.9, range = 1 to 20, [33]). In the first trapping session, 187 individuals were captured and sampled, while 175 individuals (new captures = 53, recaptures = 122) were captured and sampled in the second trapping session. The concentration of IPA (μg ml^-1^) in the blood varied markedly amongst badgers (mean = 58.2, SD = 30.0, Fig 1) suggesting variation in the quantity of bait consumed and/or in timing between consumption and capture. At their first capture a total of 213 individuals (89%) had IPA concentrations > 1 μg ml^-1^ suggesting some degree of bait consumption, while 199 (83%) had IPA concentrations > 10 μg ml^-1^ suggesting they had consumed more bait. Although > 10 μg ml^-1^ was the chosen threshold, the majority of badgers had IPA levels significantly above this; 190 badgers (79%) had IPA concentrations of > 20 μg ml^-1^, and 135 badgers (56%) had values of > 50 μg ml^-1^. The percentage of captured badgers with IPA concentrations > 10 μg ml^-1^ varied amongst social groups but was > 90% in the majority (67%) of cases (Fig 2). In recaptured animals the IPA concentration measured in the second trapping event was correlated with the value from the first event (*t*_120_ = 16.20, r = 0.83, p = <0.001; Figure B in S1 Appendix), although the absolute value was significantly lower (*t*_121_ = 9.20, p ≤ 0.001, mean difference = -16.4, 95%CI = -12.9 to -19.9). Despite the evidence of a decline in IPA concentration over time, 101/105 (96%) of badgers with a positive IPA result at the first capture (> 10 μg ml^-1^) had a positive result at the second capture event.

**Fig 3:**
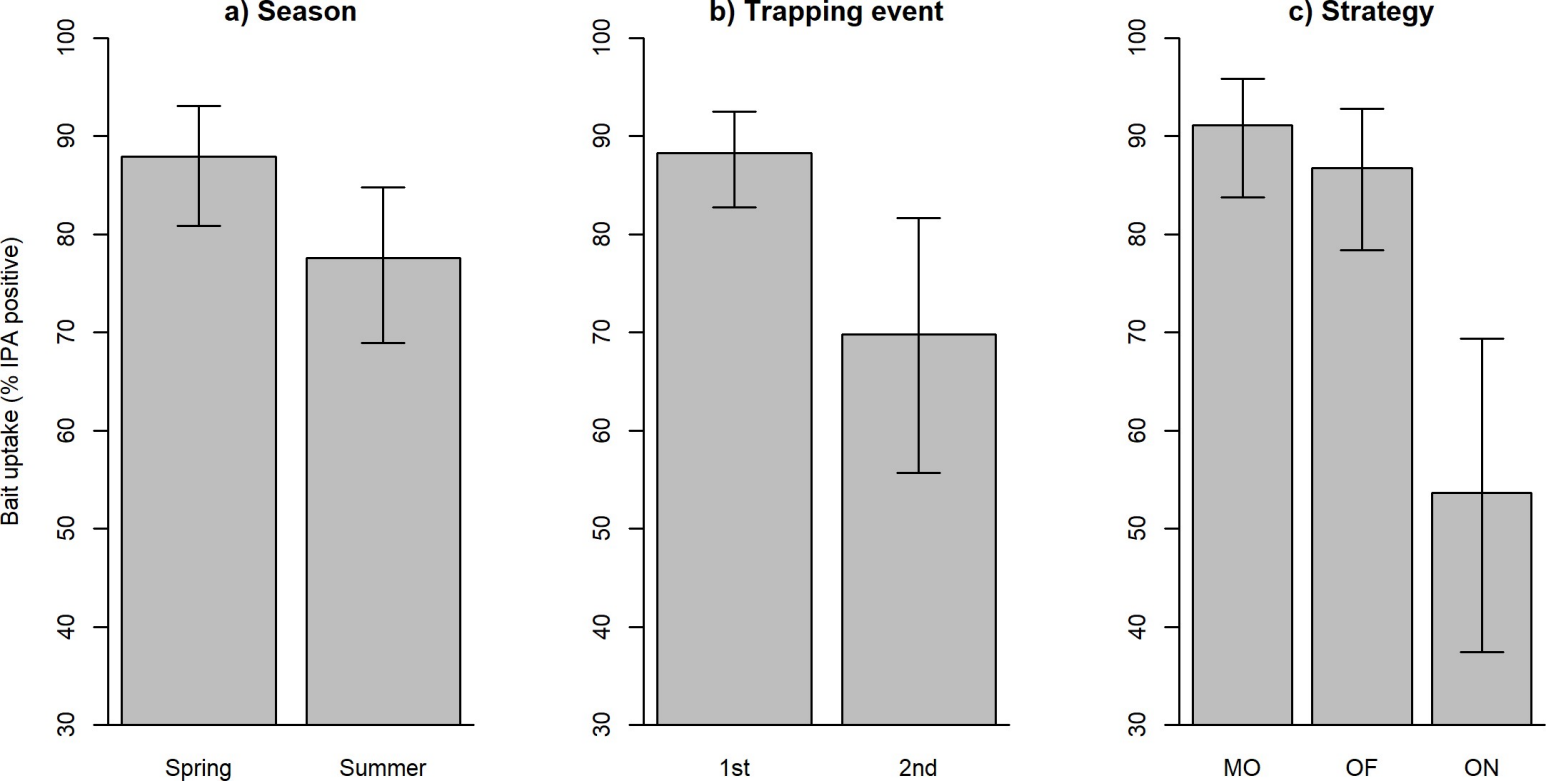
Percentage of trapped badgers with evidence of consumption of bait (as indicated by detection of >10 μg IPA in blood) in relation to season (a), trapping event (b) and deployment strategy (c). In Fig 3A, ‘MO’ = main setts only (i.e. there were no associated outlier setts), ‘OF’ = outlier setts nearby were fed along with the main setts and ‘ON’ = outlier setts nearby were not fed along with the main setts. In Fig 1B, 1^**st**^ and 2^nd^ refer to the first trapping event (10–14 days after feeding) and the second trapping event (24–28 days after feeding).

**Fig 4:**
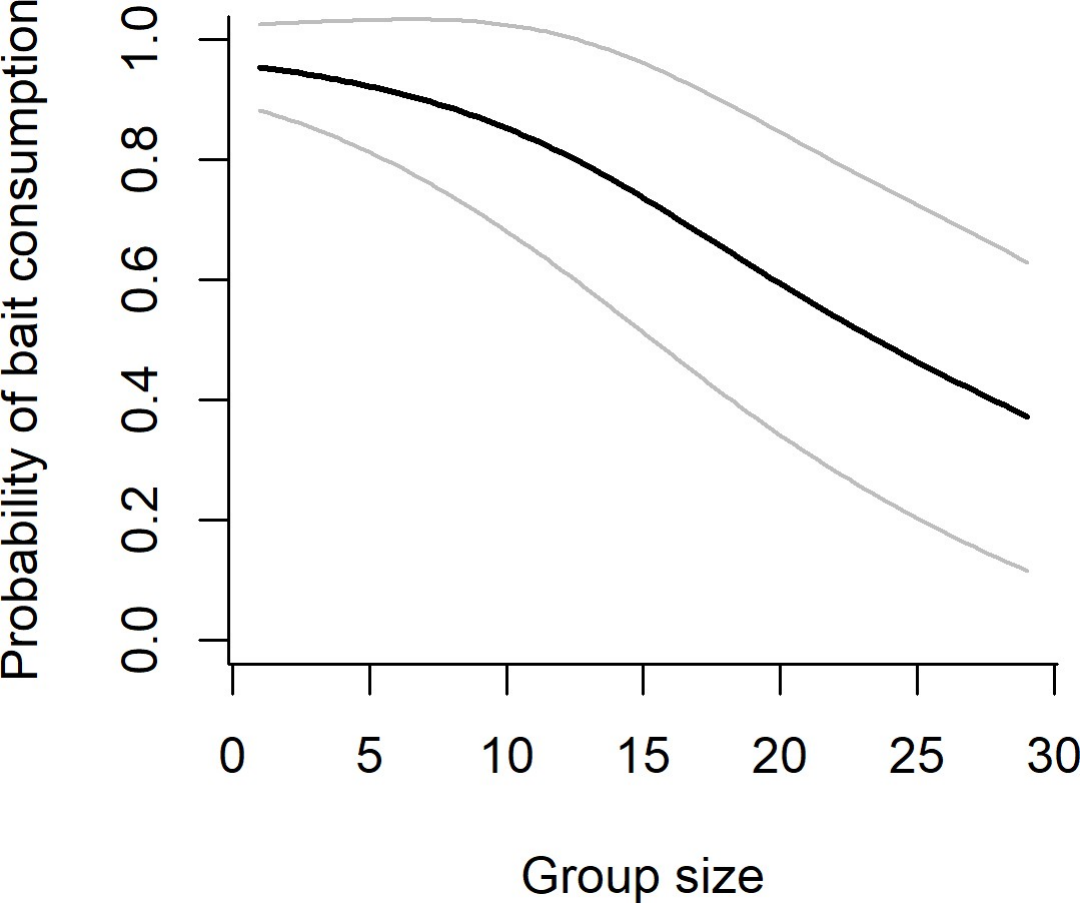
Probability of bait consumption (positive IPA result) in relation to badger group size (number of individuals captured). The black line is the marginal predicted probability from the top model explaining variation in bait consumption (grey lines show ± 1 SD).

Model averaging indicated that several variables were important predictors of bait uptake, as indicated by detection of ≥ 10 μg ml^-1^ of IPA in blood (Table 2; Table B in S1 Appendix for top model list). The probability of bait uptake was higher in badgers captured following bait deployment in spring, than in those captured following deployment in summer (Table 2, Fig 3A). The length of time between bait deployment and subsequent capture also influenced bait uptake, with a lower proportion of badgers detected as having consumed bait in the second trapping session (24–28 days after bait deployment; Table 2, Fig 3B). The bait deployment strategy also influenced uptake, with a lower probability of uptake in groups where outliers were present but not fed, than at social groups where outliers were fed (Table 2, Fig 3C). Bait uptake did not differ significantly between adults (83%) and cubs (81%). Although age, bait placement (down holes or under tiles) and an interaction between these variables were included in several top models, in each case the effects spanned zero suggesting inconsistent and non-significant effects on the likelihood of bait uptake. Finally, there was also a negative effect of putative group size on the probability of bait uptake, with lower levels of uptake at social groups where more badgers were captured and higher levels of uptake at groups where fewer badgers were captured (Table 2, Fig 4).

**Table 2:** Average model coefficients calculated for variables included in top models (AICc ≤6) explaining variation in bait uptake (IPA>10 μg ml^-1^) by captured badgers (n = 240).

Variable	Estimate	L95% CI	U95% CI	OR	Relative importance
(Intercept)	6.39	4.05	8.73	-	
**Group size**	**-0.16**	**-0.24**	**-0.07**	**0.86**	**1.00**
**Trapping session (2nd)**	**-2.12**	**-3.18**	**-1.06**	**0.12**	**1.00**
Strategy (main sett only)	-0.74	-2.18	0.70	0.48	1.00
**Strategy (outliers not fed)**	**-3.13**	**-4.76**	**-1.50**	**0.04**	**1.00**
**Season (summer)**	**-1.86**	**-3.15**	**-0.56**	**0.16**	**0.98**
Age (cub)	1.39	-0.12	2.91	4.02	0.82
Placement (above ground)	0.59	-2.42	3.60	1.80	0.67
Sex (Male)	-0.50	-1.37	0.38	0.61	0.38
Age X Placement	-1.72	-3.86	0.42	0.18	0.33
Group size X Placement	-0.22	-0.52	0.08	0.80	0.33
Area (Cirencester)	0.28	-1.99	2.55	1.32	0.28
Area (Langford)	-0.85	-2.51	0.81	0.43	0.28

Average coefficient estimates are the change in the log odds of badgers consuming baits. Odds ratios (OR) and relative importance are also displayed for each variable. Variables in bold are those with 95% confidence intervals which do not span zero indicating a consistent positive/negative effect.

## Discussion

This study confirmed that high levels of bait uptake can be achieved in wild badger populations using a formulation known to be highly palatable. Although we do not know if badgers trapped in the present study were truly representative of the wider population, and whether for example individuals that took bait were also more likely to be trapped, the results are consistent with those from previous work conducted on a well-studied population at Woodchester Park in Gloucestershire, where no such bias has been recorded [26]. Our study is encouraging for the future deployment of an oral TB vaccine for badgers [34] and contributes to our understanding of how to optimise vaccine deployment strategies, as it identifies several factors which are likely to influence levels of bait uptake in wild badgers.

The seasonal availability of natural food resources has been identified as a potential source of variation in levels of bait uptake in wildlife management studies [35], including bait delivery to badgers [27]. The present study showed that bait uptake was higher at groups fed in late spring than in those fed later in the summer, which could also be linked with temporal variation in food availability. Badgers are best described as opportunistic omnivores with earthworms (mainly *Lumbricus terrestris*) constituting the bulk of their diet in the UK [19]. As earthworms are more likely to emerge (and consequently be available to badgers) on warm, humid nights, badgers are generally expected to be more food-limited later in the summer, although in our study the highest bait uptake rates were in spring. However, short-term variation in weather conditions may influence earthworm availability on a daily basis [36] and so badger foraging behaviour, including uptake of baits, may also be subject to such fine scale temporal variation. Weather conditions in the current study were similar in the two seasons although there were more days of rainfall in summer (9–18 ml of rain over 5 days) than in spring (5–16 ml of rain over 2 days), which may have influenced our results. Alternatively, behavioural differences associated with the timing of bait provision may have played a role in the observed seasonal difference in bait uptake. High rates of bait uptake soon after cubs have weaned (May to June onwards) would be advantageous to the management of TB as this would minimise the period of time during which cubs were exposed to potential sources of infection prior to vaccination.

We found no effect of bait placement on uptake by badgers, suggesting that with the bait type and quantity used in the current study, simply placing it directly into sett entrances is as effective as dispersing baits under tiles above ground. This is encouraging for the development of bait delivery programmes as the former is a simpler and likely cheaper approach. Non-target activity was not monitored as part of this study, although previous bait deployment research indicates that a range of species may be active around badger setts and may potentially consume baits [37]. Placing baits into sett entrances also has the advantage of the bait (and ultimately a live vaccine) being largely out of reach of larger non-target species such as cattle, although consumption of baits by small rodents would still be likely [37]. However, although there was no effect of bait placement (under tiles vs down sett entrances) on uptake in badgers in the current study, reductions in the size or number of bait portions in a future bait product might increase the likelihood of intra-specific competition. In this situation, dispersing baits more widely (i.e. above ground) could be advantageous and lead to bait uptake by more group members.

Badgers have been shown to compete over supplementary food, with larger individuals typically prevailing [38]. In the current study we found no effect of age on bait uptake, suggesting that smaller cubs were able to gain equivalent access to baits as adults. However, we did detect an effect of social group size on the likelihood of bait uptake which is consistent with competition for bait. The negative correlation between bait uptake and the number of badgers trapped may have arisen because a fixed number of baits were fed at each sett and so their per-capita availability would have been greater for animals in smaller groups. These results highlight the trade-off that exists between the need to deploy a sufficient number of baits to achieve broad coverage of the population and the need to minimise costs. Ideally, the number of baits deployed would reflect the number of animals present, but estimating the number of badgers resident in a given sett is not possible from field signs [39], and the only reliable methods, such as capture mark recapture [40] or hair trapping and DNA profiling [41] are relatively labour intensive and expensive. The observed variation in biomarker levels amongst individual badgers suggests that certain animals were able to access a disproportionate amount of bait at some setts. Whilst this may not have had a significant impact on the overall level of bait uptake in this study, bait monopolisation by dominant individuals (or earlier emerging cubs) may be problematic if fewer baits are deployed [42, 43].

The present study identified a significant difference in the likelihood of a positive biomarker result depending on whether a badger was captured 10–14 days or 24–28 days after bait deployment. It is possible that this difference is partly due to a gradual decline in IPA concentration over time, as we found that recaptured animals generally had a lower concentration at the second trapping event. However, 96% of badgers with a positive result at their first capture were still positive at the second capture event, suggesting that most animals eating bait had consumed enough to maintain a high IPA concentration for a protracted time period. Variation in bait uptake between capture events could potentially relate to differences in behaviour, and if bolder individuals were more likely to consume baits they might also be over-represented in the first trapping event. Alternatively, lower levels of bait consumption by badgers caught in the second trapping event, may instead relate to the increasing likelihood of badgers moving amongst setts as time passed since the bait was fed. Studies using devices to remotely collect hairs from passing badgers for genetic analysis indicate that up to 28 days are required to hair trap all members of a social group, likely due to movement among setts [44]. This also ties in with the observation that badgers may be more likely to aggregate at main setts during the winter but make more frequent use of outlying setts during the summer [45]. In the present study social groups with outliers that were not fed bait typically had lower levels of uptake than those where outliers were fed (or where there were no outliers). Our results suggest that it may be possible to increase uptake by deploying baits over a larger area in order to target badgers at all outliers associated with a main sett, and to do so over a longer period in order to target individuals not present during the relatively short feeding period. Feeding of contiguous setts/social groups is also likely to result in higher uptake than the targeted approach that was a necessary requirement of the experimental design in this field study.

## Conclusion

An oral bait holds the best prospect for delivering vaccine to badgers over a wide geographical area and considerable resources are being directed towards the development and licensing of such a product. The present study has demonstrated that high (80–90%) rates of bait uptake may be achieved among populations of wild badgers, following the targeted deployment of a highly palatable bait at badger setts in spring/early summer. Recent studies investigating oral vaccination of badgers in Ireland suggest that uptake levels of above 30% may lead to a decline in TB in badgers. Similarly, previous studies of the effects of injectable BCG in badgers found indirect benefits (reduction in likelihood of cubs testing positive) in groups where over 30% of adults were vaccinated [14]. Future studies will be required to investigate the induced immune response of badgers that consume vaccine in baits, and the thermal stability of the vaccine within baits deployed in the environment. The present study has also highlighted differences in uptake related to whether baits were deployed at main or outlier setts, and results suggest that natural movements of badgers within the landscape may influence bait uptake, particularly if deployment is focused in certain locations or time periods. The absence of an effect of bait placement on uptake rates suggests that the simpler option of deploying bait down sett entrances may be an appropriate approach. Whilst these initial results are instructive and encouraging, this study was not able to investigate inter-annual variation in uptake rates which will need to be considered in future work. Further studies are also required to evaluate the uptake of a bait formulation [16] that will ultimately be used to deliver BCG under different deployment scenarios. The eventual cost of an oral bait containing vaccine is currently unknown, but it is likely to be considerably more expensive than the surrogate bait used in the present study. Hence the size and number of bait portions used in the present study are unlikely to reflect those of the final oral bait product. Furthermore, the impact of bait monopolisation by dominant individuals and/or interference by non-targets [16] may become proportionally more significant than indicated in this study as the quantity of bait deployed is reduced. Consequently, further research will need to explore how best to balance the impact of these competing influences in order to optimise the cost-effectiveness of a bait deployment strategy.

## Supporting information

S1 Appendix **Table A.** Treatment details for the 40 badger social groups included in the study. Groups highlighted in grey are where no badgers were captured. **Table B**. Details of top models (<6 ΔAICc) explaining variation in bait consumption. Each row represents a model and the + symbols indicate the inclusion pf variables represented by columns. **Figure A**. Number of adult badgers (dark bar) and cubs (light bar) captured, spread across the treatment categories included in the study. Figures are season (left), bait deployment (centre) and bait feeding strategy (right) where MO = main setts fed, OF = outliers present and fed, ON = outliers present but not fed. **Figure B**. Levels of IPA in blood for badgers captured during the first and second trapping events.
(DOCX)

S1 Table Raw data from sampled badgers, including IPA blood concentrations and trapping details. (XLSX)
