# Supplementary material for: Bait uptake by wild badgers and its implications for oral vaccination against tuberculosis
Source: PLoS One. 2018 Nov 9;13(11):e0206136. doi: 10.1371/journal.pone.0206136 (PMC6226152; doi:10.1371/journal.pone.0206136)
Supplement: S1 Appendix — Table A. Treatment details for the 40 badger social groups included in the study. Groups highlighted in grey are where no badgers were captured. Table B. Details of top models (<6 ΔAICc) explaining variation in bait consumption. Each row represents a model and the + symbols indicate the inclusion pf variables represented by columns. Figure A. Number of adult badgers (dark bar) and cubs (light bar) captured, spread across the treatment categories included in the study. Figures are season (left), bait deployment (centre) and bait feeding strategy (right) where MO = main setts fed, OF = outliers present and fed, ON = outliers present but not fed. Figure B. Levels of IPA in blood for badgers captured during the first and second trapping events. (DOCX) [file pone.0206136.s001.docx]

**S1. Appendix: Supporting Information**

Table A.

| **Social group** | **Season** | **Area** | **Bait Placement** | **Deployment strategy** | **n badgers caught** |
| --- | --- | --- | --- | --- | --- |
| Double Decker | Spring | Langford | Above ground | Main setts & outliers fed | 1 |
| Long Lane | Spring | Langford | Above ground | Main setts & outliers fed | 5 |
| Wilmington | Spring | Bath | Above ground | Main setts & outliers fed | 13 |
| Corner | Spring | Bath | Above ground | Main setts only | 4 |
| Riverside | Spring | Cirencester | Above ground | Main setts only | 0 |
| Swallow Cliff | Spring | Langford | Above ground | Main setts only | 5 |
| Tarlton | Spring | Cirencester | Above ground | Main setts only | 6 |
| The Mount | Spring | Langford | Above ground | Main setts only | 7 |
| Wyatt Earp | Spring | Bath | Above ground | Main setts only | 7 |
| Dock | Spring | Bath | Below ground | Main setts & outliers fed | 29 |
| Hill Fort | Spring | Bath | Below ground | Main setts & outliers fed | 8 |
| Mop | Spring | Langford | Below ground | Main setts & outliers fed | 7 |
| Sandpool | Spring | Cirencester | Below ground | Main setts & outliers fed | 3 |
| Lemonade | Spring | Langford | Below ground | Main setts fed but not outliers | 4 |
| Prior Park | Spring | Bath | Below ground | Main setts fed but not outliers | 14 |
| Dryleaze | Spring | Cirencester | Below ground | Main setts only | 1 |
| Easyjet | Spring | Langford | Below ground | Main setts only | 4 |
| Ivy Lodge | Spring | Cirencester | Below ground | Main setts only | 1 |
| Meadgate | Spring | Bath | Below ground | Main setts only | 2 |
| Tyntesfield | Spring | Langford | Below ground | Main setts only | 3 |
| Batcombe | Summer | Langford | Above ground | Main setts & outliers fed | 12 |
| Cheddar | Summer | Langford | Above ground | Main setts & outliers fed | 8 |
| Frith Wood | Summer | Cirencester | Above ground | Main setts & outliers fed | 5 |
| Hardington | Summer | Bath | Above ground | Main setts & outliers fed | 0 |
| Overtown | Summer | Cirencester | Above ground | Main setts fed but not outliers | 6 |
| Blackdown | Summer | Langford | Above ground | Main setts only | 10 |
| Chivers | Summer | Langford | Above ground | Main setts only | 9 |
| Lodge Road | Summer | Bath | Above ground | Main setts only | 5 |
| Outlands | Summer | Cirencester | Above ground | Main setts only | 0 |
| Park Wood | Summer | Bath | Above ground | Main setts only | 8 |
| Thorn | Summer | Bath | Above ground | Main setts only | 3 |
| Gruffy | Summer | Langford | Below ground | Main setts & outliers fed | 7 |
| Sandy Hill | Summer | Cirencester | Below ground | Main setts fed but not outliers | 17 |
| Big Heap | Summer | Langford | Below ground | Main setts only | 0 |
| Bridleway | Summer | Cirencester | Below ground | Main setts only | 5 |
| Nursery | Summer | Bath | Below ground | Main setts only | 4 |
| Pillar Lane | Summer | Bath | Below ground | Main setts only | 2 |
| Rams Cliff | Summer | Langford | Below ground | Main setts only | 7 |
| Rough Park | Summer | Cirencester | Below ground | Main setts only | 3 |
| Snipe | Summer | Langford | Below ground | Main setts only | 5 |
|  |  |  |  |  |  |

Table B.

| age | area | group size | trap event | placement | season | sex | strategy | age X  placement | group size X  placement | df | logLik | AICc | Δ AICc | Weight |
| --- | --- | --- | --- | --- | --- | --- | --- | --- | --- | --- | --- | --- | --- | --- |
| **+** |  | **+** | **+** |  | **+** |  | **+** |  |  | 8 | -75.72 | 168.05 | 0 | 0.09 |
| **+** |  | **+** | **+** | **+** | **+** |  | **+** | **+** | **+** | 11 | -72.68 | 168.51 | 0.46 | 0.07 |
| **+** |  | **+** | **+** | **+** | **+** |  | **+** |  | **+** | 10 | -73.84 | 168.64 | 0.59 | 0.07 |
| **+** |  | **+** | **+** |  | **+** | **+** | **+** |  |  | 9 | -75.08 | 168.94 | 0.89 | 0.06 |
| **+** |  | **+** | **+** | **+** | **+** |  | **+** | **+** |  | 10 | -74.05 | 169.06 | 1.01 | 0.06 |
| **+** |  | **+** | **+** | **+** | **+** | **+** | **+** | **+** | **+** | 12 | -71.88 | 169.13 | 1.08 | 0.05 |
| **+** |  | **+** | **+** | **+** | **+** | **+** | **+** | **+** |  | 11 | -73 | 169.17 | 1.11 | 0.05 |
| **+** |  | **+** | **+** | **+** | **+** |  | **+** |  |  | 9 | -75.22 | 169.22 | 1.16 | 0.05 |
| **+** | **+** | **+** | **+** |  | **+** |  | **+** |  |  | 10 | -74.18 | 169.32 | 1.27 | 0.05 |
|  |  | **+** | **+** |  | **+** |  | **+** |  |  | 7 | -77.52 | 169.53 | 1.47 | 0.04 |
| **+** |  | **+** | **+** | **+** | **+** | **+** | **+** |  | **+** | 11 | -73.34 | 169.84 | 1.78 | 0.04 |
| **+** |  | **+** | **+** | **+** | **+** | **+** | **+** |  |  | 10 | -74.5 | 169.96 | 1.9 | 0.04 |
| **+** | **+** | **+** | **+** |  | **+** |  | **+** | **+** |  | 12 | -72.54 | 170.45 | 2.4 | 0.03 |
| **+** | **+** | **+** | **+** | **+** | **+** | **+** | **+** |  |  | 11 | -73.7 | 170.55 | 2.5 | 0.03 |
| **+** | **+** | **+** | **+** | **+** | **+** |  | **+** |  |  | 11 | -73.75 | 170.66 | 2.6 | 0.03 |
|  |  | **+** | **+** |  | **+** | **+** | **+** |  |  | 8 | -77.05 | 170.73 | 2.68 | 0.02 |
|  | **+** | **+** | **+** |  | **+** |  | **+** |  |  | 9 | -76.08 | 170.94 | 2.88 | 0.02 |
|  |  | **+** | **+** | **+** | **+** |  | **+** |  | **+** | 9 | -76.14 | 171.07 | 3.01 | 0.02 |
|  |  | **+** | **+** | **+** | **+** |  | **+** |  |  | 8 | -77.23 | 171.08 | 3.02 | 0.02 |
| **+** | **+** | **+** | **+** | **+** | **+** | **+** | **+** | **+** |  | 13 | -71.77 | 171.14 | 3.09 | 0.02 |
| **+** | **+** | **+** | **+** | **+** | **+** |  | **+** | **+** | **+** | 13 | -71.98 | 171.56 | 3.51 | 0.02 |
| **+** | **+** | **+** | **+** | **+** | **+** |  | **+** |  | **+** | 12 | -73.14 | 171.65 | 3.59 | 0.02 |
| **+** | **+** | **+** | **+** | **+** | **+** | **+** | **+** |  |  | 12 | -73.23 | 171.83 | 3.78 | 0.01 |
|  |  | **+** | **+** | **+** | **+** | **+** | **+** |  |  | 9 | -76.72 | 172.23 | 4.17 | 0.01 |
| **+** | **+** | **+** | **+** | **+** | **+** | **+** | **+** | **+** | **+** | 14 | -71.3 | 172.46 | 4.41 | 0.01 |
|  | **+** | **+** | **+** |  | **+** | **+** | **+** |  |  | 10 | -75.77 | 172.5 | 4.44 | 0.01 |
|  | **+** | **+** | **+** | **+** | **+** |  | **+** |  |  | 10 | -75.77 | 172.51 | 4.46 | 0.01 |
|  |  | **+** | **+** | **+** | **+** | **+** | **+** |  | **+** | 10 | -75.79 | 172.54 | 4.49 | 0.01 |
| **+** | **+** | **+** | **+** | **+** | **+** | **+** | **+** |  | **+** | 13 | -72.71 | 173.02 | 4.97 | 0.01 |
| **+** | **+** | **+** | **+** | **+** |  |  | **+** | **+** | **+** | 12 | -73.98 | 173.34 | 5.29 | 0.01 |
| **+** | **+** | **+** | **+** | **+** |  |  | **+** |  | **+** | 11 | -75.2 | 173.55 | 5.5 | 0.01 |
| **+** | **+** | **+** | **+** | **+** |  |  | **+** | **+** |  | 11 | -75.31 | 173.77 | 5.71 | 0.01 |
|  | **+** | **+** | **+** |  | **+** |  | **+** |  | **+** | 11 | -75.36 | 173.89 | 5.83 | 0.01 |
|  |  |  |  |  |  |  |  |  |  |  |  |  |  |  |

Figure A.


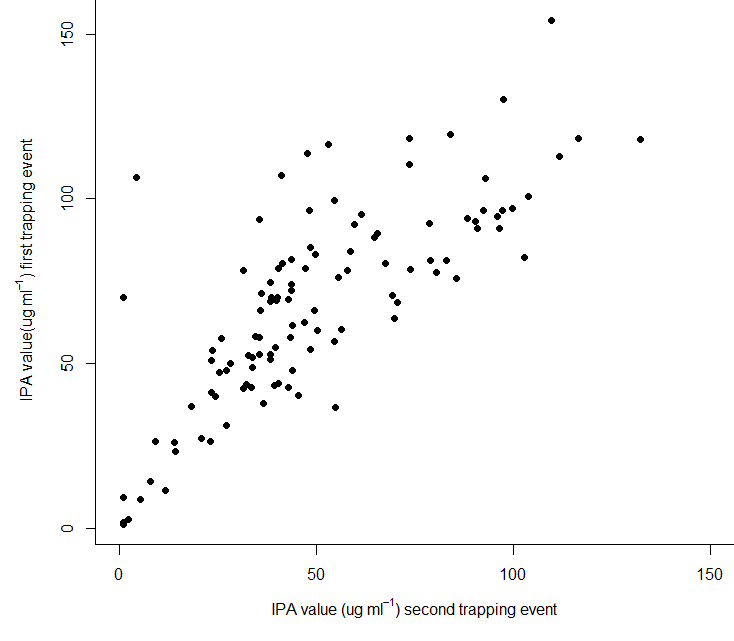


Figure B.
